# Hypoxia and mesenchymal stromal cells as key drivers of initial fracture healing in an equine *in vitro* fracture hematoma model

**DOI:** 10.1371/journal.pone.0214276

**Published:** 2019-04-04

**Authors:** Moritz Pfeiffenberger, Janika Bartsch, Paula Hoff, Igor Ponomarev, Dirk Barnewitz, Christa Thöne-Reineke, Frank Buttgereit, Timo Gaber, Annemarie Lang

**Affiliations:** 1 Department of Rheumatology and Clinical Immunology, Charité-Universitätsmedizin Berlin, corporate member of Freie Universität Berlin, Humboldt-Universität zu Berlin, and Berlin Institute of Health, Berlin, Germany; 2 German Rheumatism Research Centre (DRFZ) Berlin, a Leibniz Institute, Berlin, Germany; 3 Research Center of Medical Technology and Biotechnology, Bad Langensalza, Germany; 4 Institute of Animal Welfare, Animal Behavior and Laboratory Animal Science, Department of Veterinary Medicine, Freie Universität Berlin, Berlin, Germany; University of Edinburgh, UNITED KINGDOM

## Abstract

Fractures in horses–whether simple fractures with just one clean break, or incomplete greenstick with stress fractures, or complications such as shattered bones can all be either minimal or even catastrophic. Thus, improvement in fracture healing is a hallmark in equine orthopedics. The fracture healing process implements a complex sequence of events including the initial inflammatory phase removing damaged tissue, re-establishment of vessels and mesenchymal stromal cells, a soft and hard callus phase closing the fracture gap as well as the remodeling phase shaping the bone to a scar-free tissue. Detailed knowledge on processes in equine fracture healing in general and on the initial phase in particular is apparently very limited. Therefore, we generated equine *in vitro* fracture hematoma models (FH models) to study time-dependent changes in cell composition and RNA-expression for the most prominent cells in the FH model (immune cells, mesenchymal stromal cells) under conditions most closely adapted to the *in vivo* situation (hypoxia) by using flow cytometry and qPCR. In order to analyze the impact of mesenchymal stromal cells in greater detail, we also incubated blood clots without the addition of mesenchymal stromal cells under the same conditions as a control. We observed a superior survival capacity of mesenchymal stromal cells over immune cells within our FH model maintained under hypoxia. Furthermore, we demonstrate an upregulation of relevant angiogenic, osteogenic and hypoxia-induced markers within 48 h, a time well-known to be crucial for proper fracture healing.

## Introduction

Fractures in horses are often fatal, extremely expensive to treat, and in certain cases an injury leading to euthanasia [1, 2]. Additionally, equine fractures and their subsequent treatment are of great economic interest [3], especially for horses deployed in racing. Various studies indicated an incidence of fractures in races between 1–2% per race start [2, 4, 5], with pelvic and tibial stress fractures identified as the most common cases of fracture [5]. Furthermore, the number of horses used in leisure sports is still increasing and includes a trend towards free-range husbandries in groups leading potentially to injuries and contortions especially in the fetlock area [6, 7]. Similar to treatment in humans, current strategies towards long bone fractures in horses focus on stabilization of the fracture site by means of screws or nails [8, 9]. Nowadays more elicit methods of treatment utilizing internal fixators [1], locking compression plates [10], external fixators [11] or hydrogels [12] are being applied in clinics. Moreover, cell therapy with either mesenchymal stromal cells [13, 14] or osteoprogenitor cells [15] is of upcoming interest especially for fractures that cannot be stabilized due to the location (fetlock, coffin bone). Nevertheless, the biggest challenge still remains the appropriate stabilization that remains perpetuated during the recovery time after surgery when approximately 400–600 kg of body weight are loaded onto the bones. As to the processes of fracture healing and particularly those during the initial phase of fracture healing, only little is known. In horses, bone healing is generally considered to be delayed [16] and contradictory to phylogenetically lower developed animals, the bone quality is diminished after trauma [17].

Generally, fracture healing can be divided into four different phases: (i) initial/inflammatory phase, (ii) soft callus formation, (iii) hard callus formation, and (iv) remodelling phase. During fracture, the bone marrow channel is shattered and evading cells such as mesenchymal stromal cells (MSCs), hematopoietic stem cells (HSC), immune cells and their precursor cells are mixed with cells from ruptured blood vessels (immune cells) within the fracture gap. These cells coagulate and form the so-called fracture hematoma (FH), which initiates the ongoing inflammatory phase within a hypoxic milieu [18]. Main research progress focusing on the initial phase has been conducted in sheep or rodents [19, 20], facing the problem of translation towards the human situation and/or the horse as a patient. Mice for instance lack the Haversian canal system [21], which is typical for human and equine bone physiology and remain in general an arguable model for disease patterns with ongoing inflammation processes [22]. In contrast, large animal models show considerably more similarity to human bone physiology concomitant processes when it comes to the pathophysiology of fracture healing [23]. In a human *ex vivo* study in 2011, Hoff et al. could show that besides myeloid cells of the innate immune system (monocytes, granulocytes) and cells of the adaptive immune system (T and B cells), also hematopoietic stem cells and MSCs are prominent cells in the FH [18].

Based on the general assumption that within the initial phase of bone healing immediately following the trauma, a hematoma is generated which accumulates cells from both peripheral and intramedullary blood, as well as bone marrow cells including mesenchymal stromal cells (MSCs) [24]. The initial phase is known to involve an acute inflammatory response including the production and the release of several important molecules, such as IL6, IL8 and MIF [25], and the recruitment of MSCs in order to generate a primary cartilaginous callus [24]. Thus, we generated a blood clot with MSCs to simulate the shift from the initial hematoma to the soft callus phase and to determine the importance of MSCs in the equine fracture healing process. In brief, the rationale of our study was to study the influence of MSCs, which are considered to be the main driver of tissue regeneration during the initial phase of fracture healing, since MSCs are the progenitor cells both for cartilage (endochondral ossification) and bone cells (intramembranous ossification). We also implemented microenvironmental conditions found at the fracture site in vivo (hypoxia). For simulating the hypoxic conditions, we incubated our FH models under hypoxia (1% O_2_), with normoxia serving as a control. To underline the impact of MSCs, we also incubated in parallel blood clots without the supplementation of MSCs as a second control. This way we had the opportunity to (i) contextualize our data to existing *in vivo* and *ex vivo* data and (ii) to use this system to eventually analyze the impact of fracture healing-relevant drugs or therapies in subsequent studies. To this end, we used our *in vitro* FH model in order to study the influence of hypoxia and mesenchymal stromal cells on the initial phase of fracture healing.

## Materials and methods

### Blood samples

EDTA blood samples (residual material from diagnostic blood drawings) were obtained from the equine clinic at the Department of Veterinary Medicine, Freie Universität Berlin (clinic's own horses). The horses showed no indication of illness, systemic inflammation or infection. For the *in vitro* FH models we used the blood of three different horses and mixed them with 2.2 x 10^5^ MSCs. Correspondingly, we used the blood of three different horses for the coagulation of the blood clots. Age and gender of the respective donors can be found in Table B in S1 Appendix. Blood collection was approved by the local legal representative animal rights protection authorities (Landesamt für Gesundheit und Soziales Berlin: O 344/13)

### Bone marrow-derived MSC isolation and incubation

Bone marrow was obtained from the sternum shortly after euthanasia from horses which were euthanatized for other ethical justifiable reasons (cadavers) at the equine clinic Seeburg (Dallgow-Döberitz, Germany). Horses showed no indices of illness, systemic inflammation or infection. Bone marrow was transported aseptically in phosphate-buffered saline (PBS) and at RT. Collected bone marrow was transferred into 175 cm^2^ cell culture flasks (Greiner Bio-one, Kremsmünster, Austria) and flushed with 25 ml of DMEM plus GlutaMAX (Thermo Fisher Scientific, Waltham, USA) supplemented with 20% (v/v) StemMACS MSC Expansion Media Kit XF (Miltenyi Biotech, Bergisch Gladbach, Germany), 10% (v/v) FCS (Thermo Fisher, Waltham, USA), 100 units/ml penicillin and 100 mg/ml streptomycin (Thermo Fisher, Waltham, USA), further referred to here as MSC culture medium. Incubation was carried out at 37° C in humidified atmosphere containing 5% CO_2_. The MSC culture medium was completely replaced after two days of incubation in order to remove remaining bone marrow, blood and non-adherent cells. Hereafter the medium was replaced weekly.

### Differentiation and characterization of bone marrow-derived MSCs

To ascertain the differentiation capability, cells were plated at 1x10^4^ cells/well in 96-well plates (Greiner Bio-one, Kremsmünster, Austria) and incubated in the appropriate differentiation medium for 3 weeks.

*Osteogenesis*: MSCs were differentiated in StemMACS OsteoDiff (Miltenyi Biotech, Bergisch Gladbach, Germany). Cells were fixated with a 4% (w/v) paraformaldehyde solution (Carl Roth, Karlsruhe, Germany) for 10 min at RT and stained with 2% (w/v) Alizarin Red (in H_2_O_dd_, pH 4.1; Sigma Aldrich, St. Louis, USA) for 10 min at RT.

*Adipogenesis*: MSCs were incubated in α-MEM (Sigma Aldrich, St. Louis, USA) supplemented with 10% (v/v) human serum AB (EUROCLONE, Via Figino, Italian), 100 units/ml penicillin, 100 mg/ml streptomycin, 12 mM L-glutamine (GE Healthcare, Little Chalfont, England), 5 μg/ml insulin (Lilly, Bad Homburg, Germany), 50 μM indomethacin (Sigma Aldrich), 1 μM dexamethasone (Sigma Aldrich) and 0.5 μM isobutylmethylxanthine (Sigma Aldrich, St. Louis, USA). Cells were fixed with 4% (w/v) paraformaldehyde for 10 min at RT and stained with 0.3% (v/v) Oil Red O (Sigma Aldrich, St. Louis, USA) in 60% (v/v) isopropanol (Merck, Darmstadt, Germany) for 15 min.

Further phenotypic characterization was carried out by the expression analysis of three surface MSC markers. Antibodies against equine CD29 and CD105 were used as positive markers and an antibody against equine CD14 as negative marker. Further procedure is described below (“Flow cytometric analysis”).

### Establishment of 3D fracture hematoma model and control hematomas

For the production of one hematoma model, 100 μl of blood (collected in vacutainer tubes with EDTA) were mixed with 2.2 x 10^5^ MSCs and 100 μl of a 10 mM CaCl_2_ solution in a 96-well-plate (round bottom, Greiner Bio-one, Kremsmünster, Austria). Control hematomas (blood clots) were produced analogously without any supply of MSCs. After 30 min incubation at 37°C the blood clots (n = 4) and the FH models (n = 4) were transferred into DMEM + GlutaMAX supplemented with 10% (v/v) FCS, 100 units/ml penicillin, 100 mg/ml streptomycin, 0.2% (w/v) β-glycerophosphate (Sigma Aldrich, St. Louis, USA), 10 nM dexamethasone (Sigma Aldrich, St. Louis, USA) and 0.002% (w/v) l-ascorbic acid (Sigma Aldrich, St. Louis, USA), further referred as osteogenic differentiation medium. Hematomas/blood clots were incubated for 6, 12, 24, 48, and 72 h under hypoxia at 5% CO_2_ and 1% O_2_, balanced with N_2_. Normoxic controls were incubated at 37°C under 5% CO_2_ balanced with room-air in a humidified atmosphere (resulting in 18% O_2_) for 6, 12, 24, 48, and 72 h as well.

### RNA isolation

After incubation, coagulated hematoma models were washed with PBS and cells were separated via a cell strainer (70 μm, Corning, New York, USA). Erythrocyte lysis was performed (erythrocyte lysis buffer: 0.01 M KHCO_3_, 0.155 M NH_4_Cl, 0.1 mM EDTA, pH 7.5) for 6 min at 4°C three times, and cells were washed with 0.5% (w/v) BSA in PBS (PBS/BSA). Total RNA was extracted using Arcturus PicoPure RNA Isolation Kit (Applied Biosystems, Foster City, USA), according to the manufacturer´s instructions and the RNA concentration was determined using Nanodrop ND-1000 (Peqlab Biotechnologie, Erlangen, Germany). RNA was stored at -80°C until further processing.

### Quantitative PCR (qPCR)

The cDNA was synthesized by reverse transcription using TaqMan Reverse Transcription Reagents (Applied Biosystems) for RNA concentrations >10 ng/μl or Sensiscript Reverse Transcription Kit (QIAGEN GmbH, Hilden, Germany) for RNA concentrations ≤ 10 ng/μl. cDNA was stored at -20°C until further processing. qPCR was performed using the DyNAmo Flash SYBR Green qPCR Kit (Thermo Fisher, Waltham, USA) and the Stratagene Mx3000P (Agilent Technologies, California, USA). Initial denaturation was for 7 min at 98°C followed by 45 cycles with 5 s at 98°C, 7 s at 58°C and 9 s at 72°C. Finally, the melting curve was analyzed by a stepwise increase of the temperature from 50 to 98°C every 30 s.

All primers were purchased from TIB Molbiol (Berlin, Germany; gene symbol: forward primer, reverse primer):

***B2M***: CCCCTGATAGTTAAGTGGGATCG, AGTACAGCTTCCTGATTTATGTGC;

***MIF***: GCAAGCCAGCCCAGTACATC, GCTGTAGGAGCGGTTCTGTG;

***VEGFA***: TTGCTGCTCTACCTCCACCAT, ATCAGGGGCACACAGGAT;

***RUNX2***: TGTCATGGCGGGTAACGAT, TCCGGCCCACAAATCTCA;

***SLC2A1***: GAAACCTCACCCCACATCCT, TTCGCCTTCCGTAGTTCTCA;

***LDHA***: GCCGTCTTAATTTGGTCCAG, TGGATTGGAAACAACAAGCA;

***PFKFB3***: GATTTAGCACAAAGCACGTTT, CTCCAAGGGCATCTTCACAG;

***PGK1***: GAACACGGAGGATAAAGTCAGC, AGGAACCAAAAGGCAGGAAA;

***SPP1***: CCAGTGAGCATTCCGATGTG, TCTCCCACCCCGCTATTATTT;

***PPARG***: GGGTGTCAGTTTCGCTCAGT, GGGCTCCATAAAGTCACCAA.

Data were normalized to the expression of *Beta-2-Microglobulin* (B2M) and to the time point 0 h, using the ΔΔCt-method. Focusing on the influence of hypoxia and the effect of MSCs on a model of a fracture hematoma using equine samples under a sterile inflammatory situation, we had to exclude commonly used housekeeping genes that are known to be regulated by hypoxia or inflammation such as *GAPDH* or *ACTB*. We have ultimately chosen *B2M* as a housekeeping gene which has been reported to be a stable housekeeping gene in horse and under hypoxic conditions at least in human MSCs [26, 27]. Furthermore, using qPCR based on the same template concentrations we observed neglectable deviations of the Ct-values of *B2M* with regard to incubation duration for different time points (0 h, 12 h, 48 h) data not shown.

### Flow cytometric analysis

After erythrocyte lysis, the isolated cells were washed with PBS/BSA. After Fc-receptor blocking with Flebogamma the cells were washed with PBS/BSA and antibody staining was performed for 15 min on ice. Table A in S1 Appendix shows all antibodies with their specificity, dilution used and the corresponding isotype controls. All isotype controls were obtained from Miltenyi Biotech GmbH (Bergisch Gladbach, Germany). The cells were washed with PBS/BSA and incubated with 1:25-diluted 7-AAD (BioLegend, San Diego, USA) for 15 min at RT. After a further washing step with PBS/BSA, the cells were resuspended in 0.05% (w/v) NaN_3_ in PBS/BSA (PBS/BSA/Azide). The cells were recorded using flow cytometry with a MACS Quant Analyzer (Miltenyi Biotech, Bergisch Gladbach, Germany) and analyzed with FlowJo software (Tree Star, USA). The antibodies and gating strategy utilized are given in the supplementary files (Table A and Fig A in S1 Appendix).

### Embedding, cryosections and DAPI stain

For immunofluorescence, FH models (0 h, 24 h and 48 h) were embedded as follows: FH models were transferred into 4% paraformaldehyde, then into a 10%, 20% and finally 30% glucose solution, each for 24 h. Storage was at 4°C. Cryo-embedding was followed by cryosections as described previously [28]. Slides were air dried and subsequently stained with DAPI. The DAPI staining solution was 0.1% (v/v) DAPI (Sigma Aldrich, St. Louis, USA); 0.1% (v/v) Tween 20 (Carl Roth, Karlsruhe, Germany); 5% (v/v) FCS (Thermo Fisher, Waltham, USA) in PBS. The whole procedure was performed at RT. Sections were first incubated in PBS with 0.1% (v/v) Tween 20 for 10 min. After 10 min incubation in DAPI staining solution, the sections were washed three times in PBS with 0.1% (v/v) Tween 20. Stained sections were put on a slide and then mounted (Fluoromount™ Aqueous Mounting Medium, Sigma Aldrich, St. Louis, USA) under a cover slip. Examination of the sections was performed and photos were taken, using a KEYENCE BZ-X700 fluorescence microscope and depicted in pseudo-colors.

### Statistical analysis

Statistical tests were performed using Graph Pad Prism Software (La Jolla, USA). Differences were compared using the Mann–Whitney U-test. Probability values of p<0.05 were considered to be statistically significant, and values of p<0.1 were considered to have a statistical trend (*p<0.05; ^+^p<0.1).

## Results

**Fig 1:**
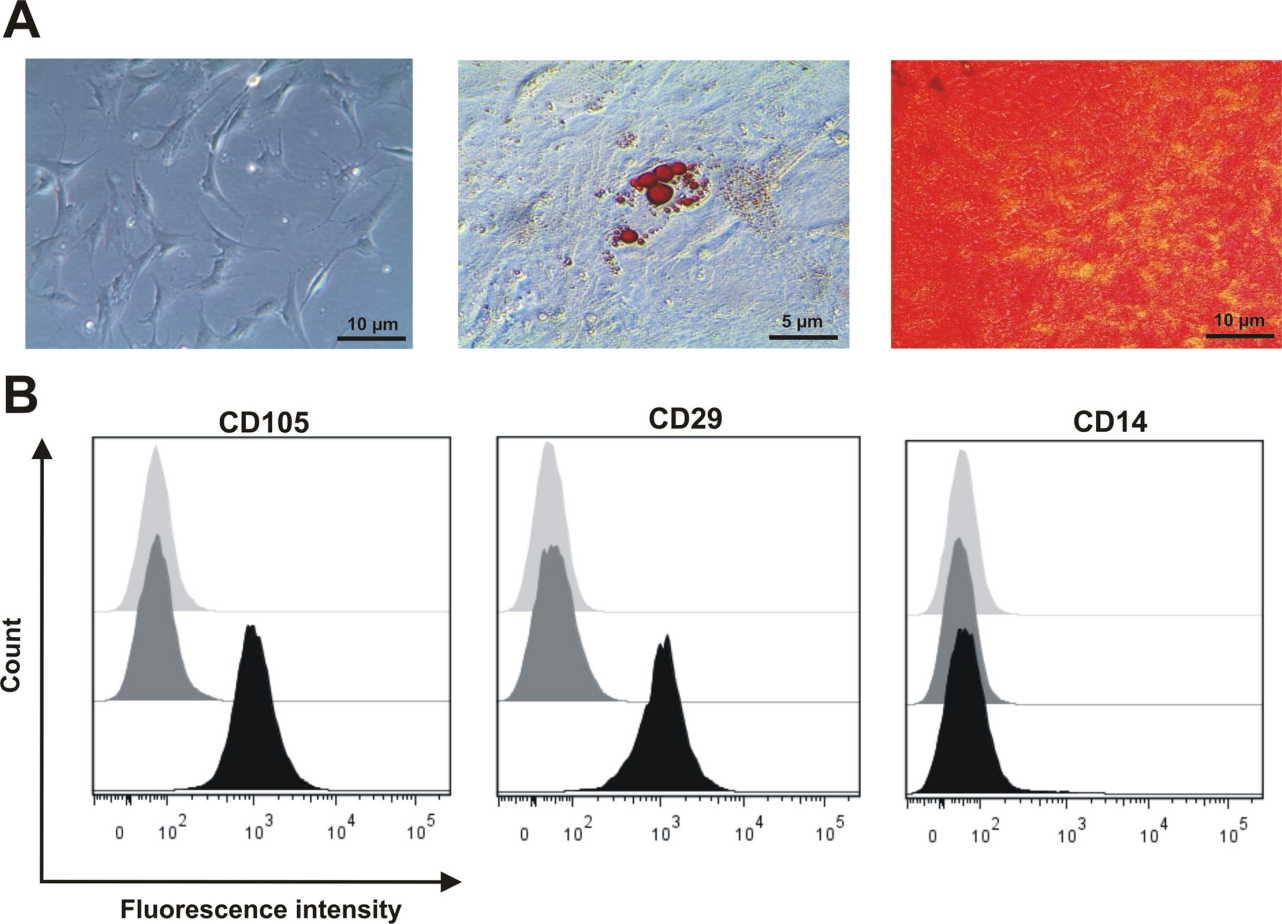
Bone marrow-derived cells obtained from sternum-biopsies are characterized as MSCs. Characterization of equine bone marrow-derived MSCs obtained from sternum-biopsies with regard to their potential to (A) adhere to plastic surfaces and typical morphology, their differentiation potential towards adipogenic and osteogenic lineage and (B) express surface markers CD29, CD105, and CD14 (unstained fractions, isotype control stainings and antigen-specific stainings are depicted in light grey, dark grey and black, respectively).

MSCs play a fundamental role in the initial phase of fracture healing. Therefore, MSCs represent an important cell fraction within our hematoma models. For the use of MSCs, we established well-defined minimal criteria based on their potential to adhere to plastics, to differentiate into osteoblasts and adipocytes, and to express typical surface markers. Only MSCs that fulfill these criteria were utilized to establish the equine *in vitro* FH model, consisting of peripheral blood and MSCs.After cultivation for three passages, the MSCs adhered to the plastic surface and showed their typical fibroblastoid morphology (Fig 1A). They also could be differentiated into the osteogenic lineage as the Alizarin Red S staining showed calcium-complexes stained in red colour and the adipogenic lineage as the cells secrete lipid droplets which are stained red via Red oil staining (Fig 1B). Additionally, the typical surface markers CD29 and CD105 were expressed with no expression of the exclusion marker CD14 (Fig 1C; Table A in S1 Appendix).

### Cell composition of *in vitro* FH models under normoxic conditions

**Fig 2:**
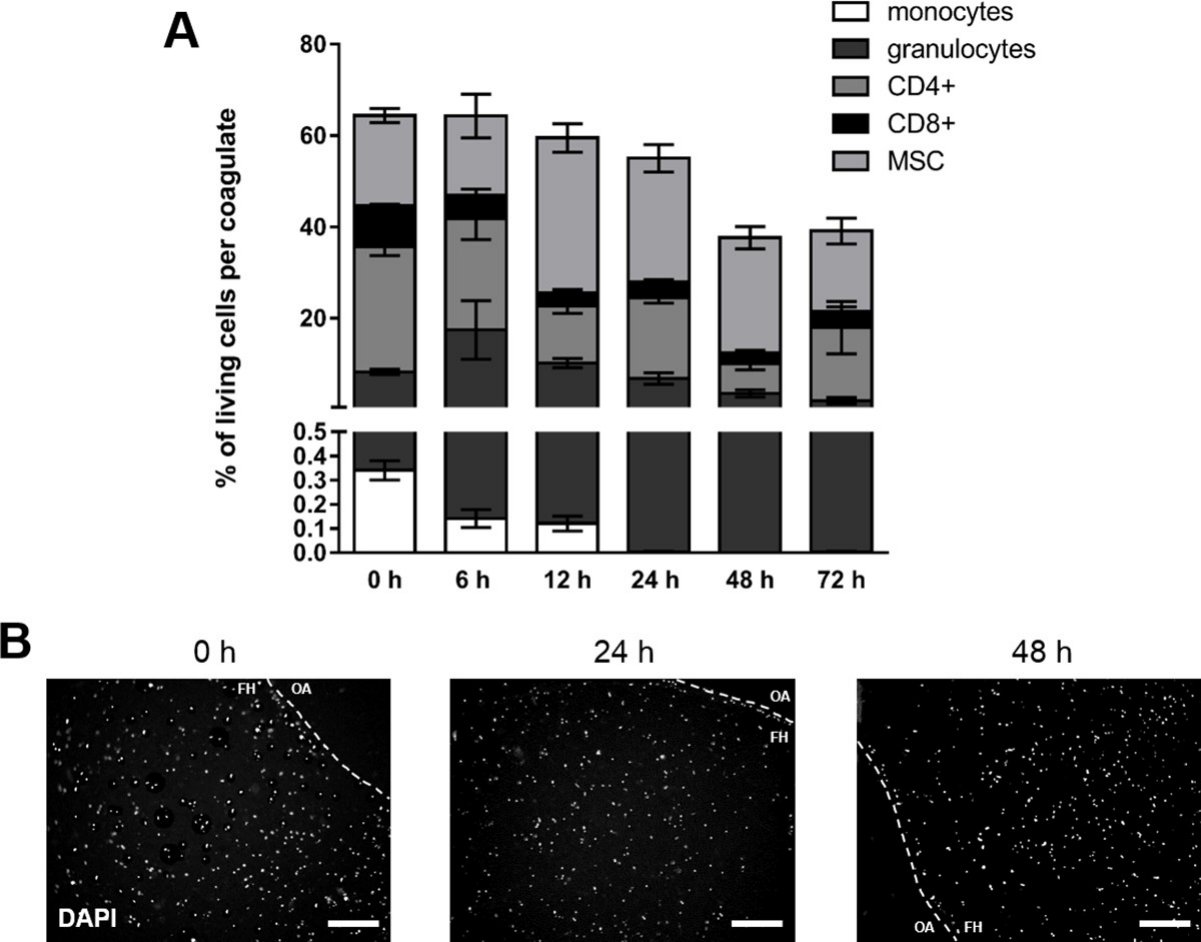
Immune cell vitality in the FH model decreases over time irrespective of subpopulation and spatial distribution after incubation under normoxic conditions. (A) Frequency of immune cell populations (granulocytes, CD14+ monocytes, CD4+ T cells, CD8+ T cells) and MSCs (CD29+, CD 105+, CD14-) negative for 7-AAD present in the *in vitro* FH model as incubated in osteogenic differentiation medium under normoxic conditions (37°C, 5% CO_2_, 18% O_2_) for 6, 12, 24, 48, and 72 h (mean ± SEM, n = 3). Depicted is the frequency of total cells and the corresponding frequencies of the cell populations. (B) Spatial distribution of cells within the *in vitro* FH model as determined by DAPI-staining and depicted as representative staining for incubation periods of 0, 24, and 48 h (The dotted line indicates the border of the *in vitro* hematoma; FH = area of the fracture hematoma model and OA = outer area).

To mimic the initial phase of fracture healing *in vitro*, we generated *in vitro* FH models by mixing and coagulating MSCs and blood cells. After incubation of the *in vitro* FH models for 6, 12, 24, 48, and 72 h under normoxic conditions (37°C, 5% CO_2_, 18% O_2_), we observed a continuous decline in the frequency of cells alive which resulted in 45 ± 3% of cells alive after an incubation period of 72 h (Fig 2A). Within the FH model, the frequency of immune cells decreased over time, while the frequency of MSCs increased within the first 12 h. As a result, the MSC population became the major cell population in the *in vitro* FH model, although it decreased between 12 and 72 h (Fig 2A). With regard to the proportion of immune cells, we observed a continuous decrease in the frequency of granulocytes over time. The frequency of monocytes was negligible, with almost no cells detectable after 6 h of cultivation, while the frequency of CD8+ cells also decreased perpetually. Interestingly, the most prominent population at 0 h–namely CD4+ T cells–remains the most stable cell population within the incubation period analyzed (Fig 2A). In contrast, with regard to the blood clots (Fig B in S1 Appendix) the frequency of granulocytes as well as T cells was very stable. As to the spatial distribution, we observed no clustering of cells but an even distribution within the FH model using DAPI-staining (Fig 2B).

### Cell composition of *in vitro* FH models under hypoxic conditions

**Fig 3:**
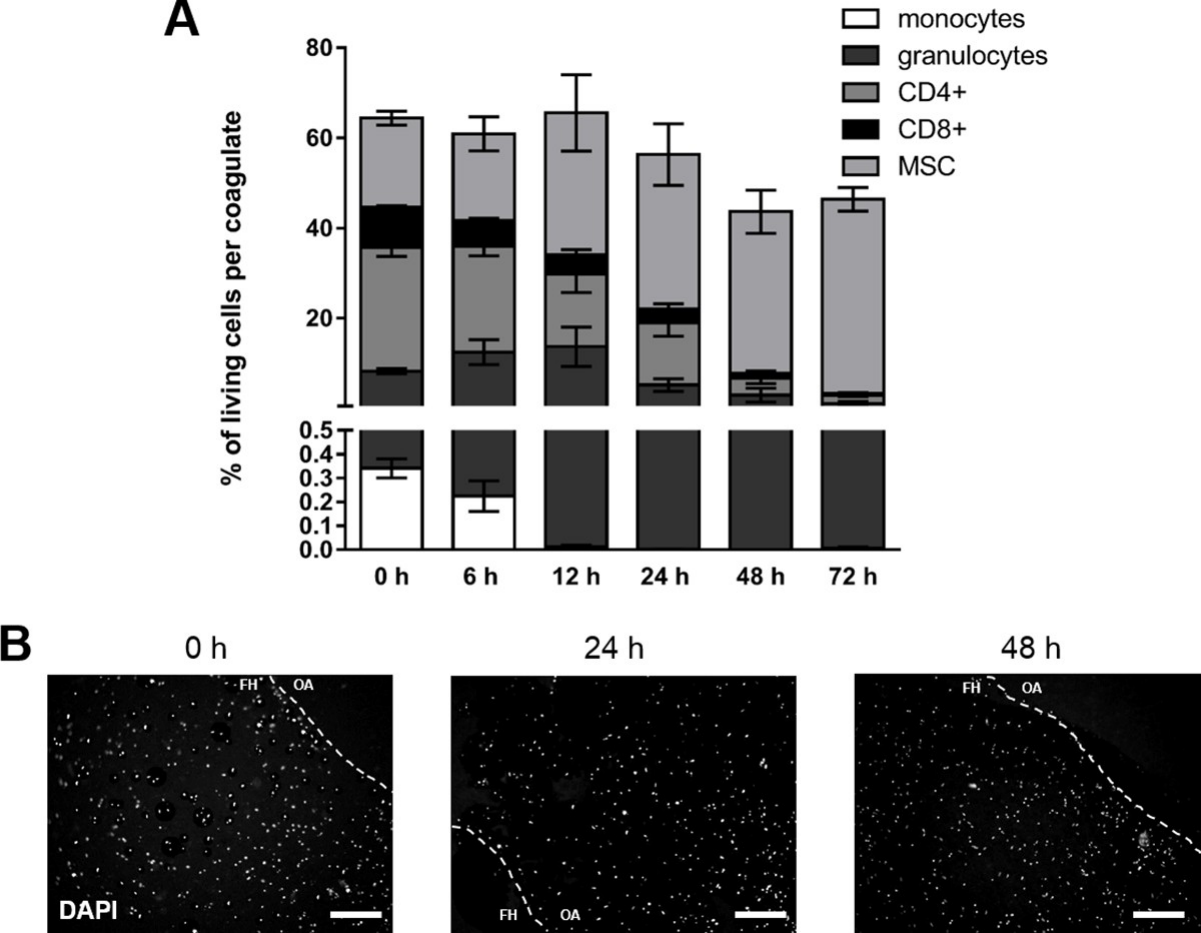
Hypoxia favors survival of MSCs while decreasing immune cell fractions. (A) Frequency of immune cell populations (granulocytes, CD14+ monocytes, CD4+ T cells, CD8+ T cells) and MSCs (CD29+, CD 105+, CD14-) negative for 7-AAD in the FH models cultured in osteogenic differentiation medium under hypoxic conditions (37°C, 5% CO_2_, 1% O_2_) for 6, 12, 24, 48, and 72 h (mean ± SEM, n = 3). Depicted is the frequency of total cells and the corresponding frequencies of the cell populations. (B) Spatial distribution of cells within the FH model as determined by DAPI-staining and depicted as representative staining for 0, 24, and 48 h of incubation (The dotted line indicates the border of the *in vitro* hematoma; FH = area of the fracture hematoma model and OA = outer area).

To mimic the restricted microenvironment in the initial phase of fracture healing more adequately, we generated *in vitro* FH models and incubated them under hypoxic conditions (37°C, 5% CO_2_, 1% O_2_) for 6, 12, 24, 48, and 72 h. We observed a perpetual decline of the cells alive with a final frequency of 43 ± 2% after 72 h of incubation (Fig 3A). With regard to the immune cell populations, the frequency of CD4+ T cells and CD8+ T cells again constantly decreased over time, whereas the frequency of granulocytes increased from zero to 12 h before massively decreasing. The frequency of monocytes alive was barely detectable at any time point analyzed. Finally, none of the analyzed immune cell populations survived the FH model after 72 h of cultivation under hypoxic conditions. In contrast, the proportion of MSCs within the FH model permanently increased from a ratio of 20% ± 1% at 0 h to 42 ± 2% at 72 h after cultivation (Fig 3A). With regard to the blood clots (Fig B in S1 Appendix), we observed a stable frequency of granulocytes and T cells. Regarding the spatial distribution, we again observed no clustering of cells but an even distribution within the FH model using DAPI-staining (Fig 3B).

### Impact of oxygen availability on cellular vitality and composition in the *in vitro* FH models

**Fig 4:**
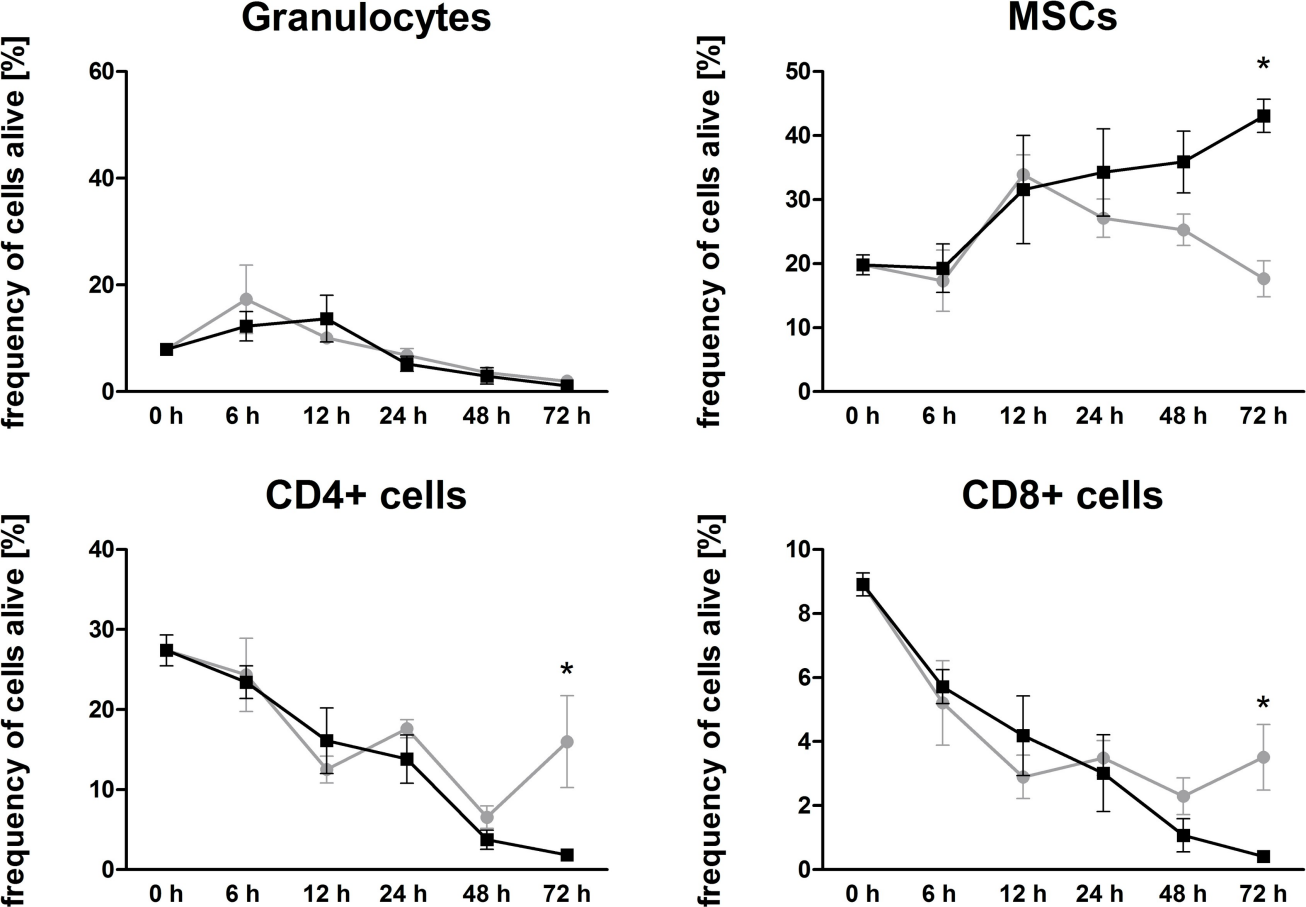
Hypoxia favors survival of MSCs while decreasing granulocytes, CD4+ and CD8+ immune cells. Frequency of granulocytes, CD4+ and CD8+ immune cells negative for 7-AAD in the FH model cultured in osteogenic differentiation medium under either normoxic (grey) or hypoxic (black) conditions (n = 3). Statistical analysis was conducted using the Mann-Whitney U-test, *p<0.05.

When comparing cellular vitality and composition of the FH models incubated under normoxia with those incubated under hypoxia, we observed no differences in respect to overall cell survival, either after 72 h, or throughout the whole decline in time. However, as far as the cellular composition is concerned, the frequencies of all immune cell populations in the FH model declined under hypoxia, whereas under normoxia, survival of CD4+ and CD8+ T cells after 72 h of incubation was significantly higher than that under hypoxic incubation (Fig 4). In contrast, the frequency of granulocytes remains unaffected by the incubation conditions, and for monocytes the case was likewise negligible. Hence, the frequency of MSCs was significantly higher under hypoxic conditions after 72 h of cultivation when compared to that of the corresponding control (Fig 4).

MSCs along with their time-dependent increase were by far the most abundant cells, a most striking situation seen from 12 to 72 h. Compared to normoxic conditions, MSCs survived better under hypoxic conditions, whereas immune cells seem to have a diminished survival rate (Fig 4).

### Time-dependent RNA-expression of fracture-healing-relevant markers in the *in vitro* FH models

**Fig 5:**
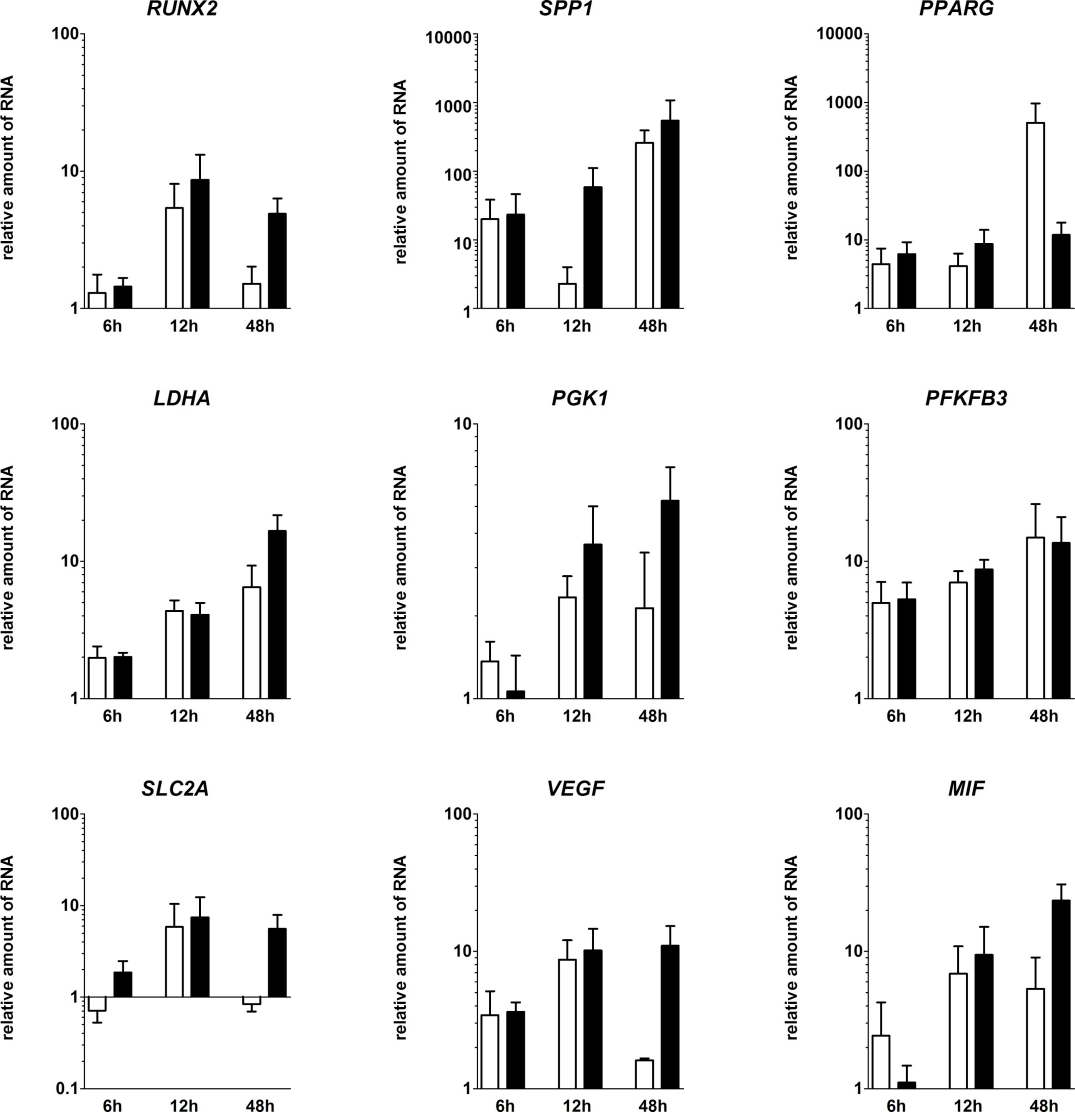
Within the FH models, osteogenic, hypoxia-induced and angiogenic markers were upregulated over time until 48 h of incubation. Depicted is the relative RNA-expression of the osteogenic markers *RUNX2*, *SPP1*, the adipogenic marker *PPARG*, the hypoxia induced genes *LDHA*, *PGK1*, *PFKFB3*, *SLC2A1*, and the angiogenic genes *VEGFA*, and *MIF* within the *in vitro* FH models after cultivation in osteogenic differentiation medium for 6, 12, and 48 h under either normoxia (white bars) or hypoxia (black bars). All values are normalized to the “housekeeping gene” *B2M* and 0 h (Mean ± SEM, n = 3). Statistical analysis was conducted using Mann-Whitney U-test.

To analyze the impact of hypoxia on all cells in the FH model, we focused on markers for osteogenesis, glycolytic adaptation towards hypoxia and angiogenesis on the transcriptional level (Fig 5). Therefore we analyzed the RNA-expression of fracture-relevant markers. We cultivated the *in vitro* hematomas for 6, 12 and 48 h. Within the hematomas, osteogenic (*RUNX2*, *SPP1*), angiogenic (*VEGFA*, *MIF*) as well as hypoxia-induced (*LDHA*, *PGK1*, *PFKFB3*, *SLC2A1*) markers were higher expressed after 48 h. Concerning the osteogenic markers, we demonstrated a time-dependent upregulation of *RUNX2* and *SPP1*. While *RUNX2* was upregulated after 12 h of cultivation, the highest upregulation of *SPP1* was observed at 48 h. Additionally, this effect was even stronger under hypoxic conditions, where *RUNX2* was upregulated to a higher extent. In contrast, the adipogenic transcription factor *PPARG* was less strongly induced in FH models incubated under hypoxic conditions. Drawing the focus towards angiogenic markers, we could show the upregulation of typical markers (peaks: *VEGFA* after 12 and *MIF* after 48 h). Interestingly, both *VEGFA* and *MIF* showed higher levels of expression in FH models incubated under hypoxic conditions. *LDHA*, *PGK1*, *PFKFB3* and *SLC2A1* were also upregulated at least after 12 h. After 48 h, the majority of genes analyzed were expressed to a higher extent under hypoxic conditions except for *PFKFB3* and *PPARG* (Fig 5).

### Differences of the mRNA-expression of *in vitro* hematomas versus blood coagulates after 48 h of cultivation under normoxic or hypoxic conditions

**Fig 6:**
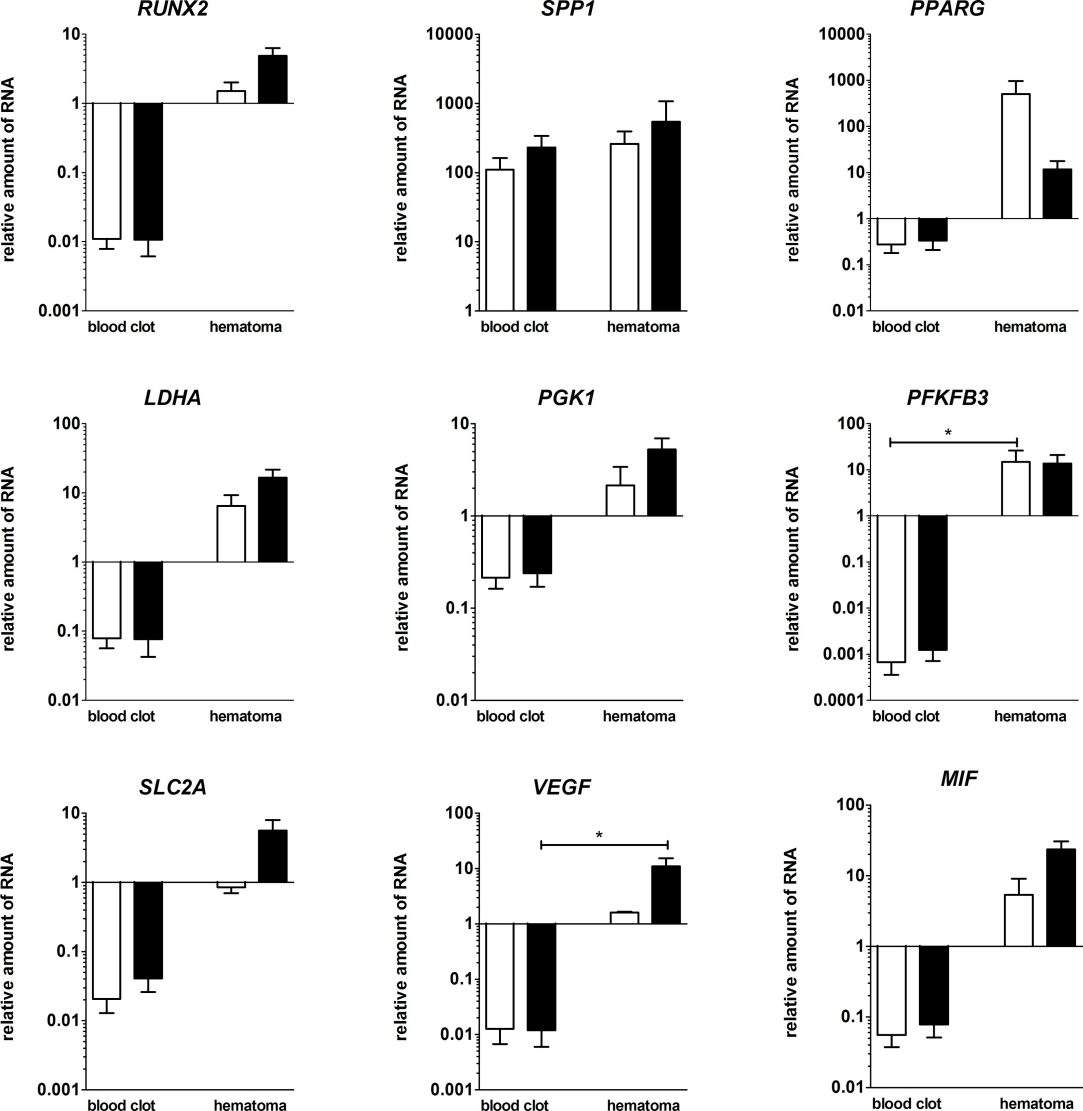
Osteogenic, hypoxia-induced and angiogenic markers were upregulated in the *in vitro* FH models compared to blood coagulates. Depicted is the relative RNA-expression of the osteogenic markers *RUNX2*, *SPP1*, the adipogenic marker *PPARG*, the hypoxia induced genes *LDHA*, *PGK1*, *PFKFB3*, *SLC2A1* and the angiogenic genes *VEGFA* and *MIF* within the FH models and the blood-only coagulates after cultivation in osteogenic differentiation medium for 48 h under either normoxic (white bars) or hypoxic conditions (black bars). All values are normalized to the “housekeeping gene” *B2M* and to 0 h (mean ± SEM, blood clots: n = 4, FH models: n = 3). Statistical analysis was conducted using Mann-Whitney U-test, *p<0.05.

To analyze if the observed changes in gene expression patterns are due to the different survival rates of the analyzed immune cell populations and MSCs, we compared the data gained from *in vitro* hematomas to blood coagulates without MSCs. Focusing on the differences between the mRNA-expression of blood coagulates and *in vitro* hematomas, we could show that except for *SPP1* the expression of relevant genes was higher in the *in vitro* hematomas (significant for *PFKFB3* and *VEGFA*). In the *in vitro* FH model, osteogenic-relevant genes (*RUNX2*, *SPP1*) were upregulated, even more pronounced under hypoxic conditions, whereas the adipogenic marker *PPARG* was upregulated, but about 100 times less under hypoxic conditions. Hypoxia-induced genes (*PGK1*, *PFKFB3* and *SLC2A1*) were also upregulated under both cultivation methods, except for *SLC2A1* which was only upregulated under hypoxic conditions. *PGK1* and *SLC2A1* showed a higher expression under hypoxic conditions. For the angiogenic markers *VEGF*A and *MIF* as well as for the pH-regulating marker *LDHA*, we could show an upregulated expression which was even more evident under the influence of hypoxia (Fig 6).

## Discussion

To study the underlying cellular mechanisms of the initial inflammatory phase of fracture healing in horses, we aimed to establish an equine *in vitro* FH model. To this end, we first created protocols for the isolation and characterization of major cell types involved in the generation of the FH model, namely immune cells–released from the ruptured vessels and from the bone marrow–and mesenchymal stromal cells (MSCs) from bone and bone marrow. The limited availability of suitable antibodies for immunological characterization but also the limited knowledge of lineage markers for equine cells especially for equine MSCs belong to the challenging issues we wanted to address in the study presented here.

To characterize MSCs, standardized criteria for human material have already been established and defined more than ten years ago [29]. In brief, human MSCs have to fulfill minimal requirements including attachment to plastic surfaces, the capacity to differentiate into multiple lineage [30], as verified by differentiation towards osteogenic, chondrogenic and adipogenic lineage, and finally the presence or absence of typical surface markers (CD73+, CD90+, CD105+; CD45-, CD34-, CD14-, CD19- and HLA-DR-) [29]. Focusing on equine MSCs, it is disparately more difficult to characterize these cells in a similar way. Although a variety of characterization procedures have already been described [31–34], only few surface markers are available for immunophenotyping due to the limited availability of suitable antibodies. Here, we characterized MSCs by demonstrating (i) plastic adherence, (ii) differentiation towards osteogenic (demonstrated by Alizarin Red staining) and adipogenic (demonstrated by Red Oil staining) lineage, and (iii) immunological characterization using CD105 and CD29, previously described as stable equine MSCs markers and widely used to characterize equine MSCs [35–38] and CD14 as a negative marker (Fig A and Table A in S1 Appendix). These have also been previously demonstrated to characterize equine monocytes (2013) [39]. We defined this characterization procedure as a minimum criterion for the use of equine MSCs within our study.

For the characterization of equine immune cells, we used antibodies against CD4, CD8, and CD14, while granulocytes were determined by granularity and size using flow cytometry (Table A in Appendix 1). Separation of MSCs from the immune cell populations in the FH model was achieved by flow cytometry (Fig A in S1 Appendix).

Although *in vitro* FH models were generated in a standardized manner with regard to cell numbers and incubation times, we had to face limitations in terms of low cell recovery from coagulates after incubation, variations in hematoma size after coagulation and a massive impact on cell survival after incubation (Figs 2 and 3).

Analyzing the impact of a restricted microenvironment by focusing on hypoxia in our *in vitro* model, we did not observe any difference between normoxia and hypoxia concerning overall cell survival (Figs 2 and 3). However, we did observe a shift in the distribution of cell population towards an increase in the proportion of MSCs after incubation under hypoxic conditions (Fig 3). We assume that MSCs, well-known key players in the process of fracture healing [40–42], may promote the termination of the inflammatory phase especially in a hypoxic microenvironment, and this may resemble the *in vivo* situation at the fracture site [18]. Thus, MSCs can be assumed to be likely suitable candidates in cell-based therapeutic strategies to overcome fracture healing disorders in horses and humans [13, 14]. Additionally, we had observed previously that the osteogenic differentiation of human MSCs is enhanced in a hypoxic microenvironment but also that it does not influence cell survival or proliferation [43]. Conversely, Ranera et al. (2012) demonstrated that hypoxia limits the proliferation of equine MSCs in 2D cultures [34]. Whether or not these differences are species-specific needs to be clarified.

Focusing on myeloid cells such as monocytes and polymorph nuclear cells (PMNC), we observed a time-dependent decline of their frequencies within the *in vitro* FH model without any significant impact of hypoxia on cell survival (Figs 2–4). In general, the role of neutrophils with regard to fracture healing has to our knowledge been poorly investigated or is discussed controversially, although granulocytes are the most abundant cells in the early FH [44]. Groogard et al. (1990) reported that neutropenia in mice did not have any significant effect on fracture healing [45]. In addition, Chung et al. (2006) observed a slight increase of bony trabeculae when treating young rats with neutrophil-neutralizing antiserum in a growth plate injury model [46]. In the human *in vitro* FH model, which was performed recently from our group neutrophils seem to only marginally influence fracture healing, while being time-dependently depleted in the FH model, and even more strongly so under hypoxic conditions [47]. In contrast, a study of Kovtun et al. (2016) reveals a crucial role of neutrophils in bone healing [44]. They drastically reduced the number of neutrophils using a Ly-6G antibody in fractured mice and observed impaired bone healing after 21 d, with diminished bone content as well as impaired mechanical properties, implicating the important role of neutrophils in the very early phase of fracture healing [44]. With regard to PMNCs in our model, we could not determine any impact of hypoxia on the frequency of granulocytes (Figs 2–4). Neutrophils are primarily responsible for the removal of debris and spoilt cells in accordance with attracting monocytes to the fracture site [48] in the very early inflammatory phase, while their importance seems to be diminished in the ongoing process of fracture healing. In our study here, we could show the presence and survival of granulocytes within the first hours as well as their time-dependent decline of their frequencies within the *in vitro* FH models, which may resemble the *in vivo* situation (Figs 2–4). Although the frequency of granulocytes is time-dependently diminished, we cannot exclude, that granulocytes in apoptosis or netosis or degranulation processes have an influence on the ongoing process of fracture healing.

Among the adaptive immune cell populations, we observed a significantly enhanced hypoxia-mediated reduction in the frequencies of CD4+ and CD8+ cells after incubation of FH models for 72 h (Figs 2–4). In the line of our observation, reduction of an adaptive immune response has been reported to accelerate during bone healing [49, 50]. In more detail, Toben et al. demonstrated an accelerated fracture healing in recombination activating gene 1 knockout (RAG1(-/-)) mice lacking the adaptive immune system [49]. Although it has been demonstrated that proliferation of CD8+ cells is diminished under hypoxic conditions [51], depletion of CD8+ T-cells in a mouse osteotomy model further has been reported to enhance fracture healing [50]. These findings and our findings presented here indicate that the continuous depletion of CD8+ T cells within the fracture site seems to be a feature beneficial for the fracture healing process. However, in an *ex vivo* human FH model, we demonstrated a decrease in lymphocyte survival after 24 h–independently of oxygen availability–although the frequencies of lymphocytes after hypoxic incubation increase. The latter can be explained by the relatively decreased granulocyte proportions after 24 h of incubation under hypoxia. Interestingly, we could detect a relevant expression of active caspases indicating apoptosis in granulocytes only after 24 h of incubation under hypoxia. However, human lymphocytes from ex vivo human FH models did express active caspases under all incubation conditions but with the highest expression after 24 h under hypoxia which is in the line with the findings in the equine FH model after 48 h [47].

Moreover, it has been well demonstrated that MSCs exhibit immunosuppressive functions [52], and that they are immunotolerant [53] and known to inhibit the proliferation of T-lymphocytes [42, 52, 54–56]. Here, we demonstrate that the increase in the frequency of MSCs over time is associated with a decrease of T-lymphocytes when investigating the incubation of these cells under hypoxia. This anti-correlative development may indicate that MSCs are limiting the initial inflammatory hypoxic phase of fracture healing in a human *ex vivo* FH model and also in an equine *in vitro* FH model.

In view of the impact of hypoxia on the expression of selected genes in our equine *in vitro* FH model, we observed that upregulation of osteogenic (*RUNX2*, *SPP1*), hypoxia-induced (*PGK1*, *LDHA*, *PFKFB3*, *SLC2A1*) and angiogenic (*VEGFA*, *MIF*) genes/factors essential for fracture healing after incubation for 48 h takes place at a higher extent under hypoxic than under normoxic conditions (Fig 5). In this line of observation, we could previously demonstrate a time-dependent increase in the expression of the osteogenic *RUNX2* and *SPP1* in a human *ex vivo* FHs [18]. Although we demonstrate an increase in the expression of *PPARG (*a key marker for the adipogenic differentiation of MSCs under normoxia), its upregulation is abolished under hypoxia. Thus, hypoxia seems to shift MSCs into the osteogenic lineage, as demonstrated previously [43].

To analyze the impact of MSCs, we compared the *in vitro* generated FH models (blood coagulates with MSCs) with blood coagulates without MSCs, focusing on the expression of selected genes (Fig 6). In general, MSCs contribute either directly or indirectly to the induction of gene expression from osteogenic (*RUNX2*, *SPP1*), hypoxia-induced (*PGK1*, *LDHA*, *PFKFB3*, *SLC2A1*) and angiogenic (*VEGFA*, *MIF*) factors essential for fracture healing. The contribution of MSCs in the equine *in vitro* FH models demonstrate a similar pattern of expression with regard to the hypoxia-induced genes *LDHA*, *PGK1* and *SLC2A1* after 48 h when compared to in human *ex vivo* as well as *in vitro* studies [18, 47]. The induction of these genes especially under hypoxia may reflect the adaptation towards a hypoxic environment and the shift towards anaerobic glycolysis [57–60]. All hypoxia-related genes were to a great extent more highly expressed in the *in vitro* hematomas when compared to blood coagulates, indicating the huge influence of MSCs on the expression of hypoxia-induced genes/factors in the FH models, which also involves the gene expression of *VEGFA* and *MIF* coding for angiogenic factors. *VEGFA* is known to be expressed locally by pre-osteoblasts [61] and MSCs, and also plays an important role in the fate of MSCs towards either adipocyte or osteoblast lineage [61, 62]. Several studies identified *VEGFA* as a key factor in osteogenesis as well as in angiogenesis [63–65]. Upregulation of *VEGFA* in our model may indicate the initiation of fracture healing-relevant processes involving angiogenesis and vascularization. *MIF* is also an essential molecule for fracture healing [25, 66] and several knock-out rat models have underlined the importance of *MIF*, demonstrating a delayed fracture healing in the absence of *MIF* [67, 68]. *MIF* is secreted by MSCs [69] and is known to promote their survival [70].

Taken together, we observed in our present equine *in vitro* FH model profound similarities to our previously published results derived from a human *in vitro* FH model. These concern the expression of genes analyzed for the upregulation of angiogenic and hypoxia-induced markers, and the indication that they are more pronounced under hypoxic conditions [47].

## Conclusion

In our study presented here, we characterized equine immune cells as well as MSCs and used these cells to establish an equine *in vitro* FH model. We demonstrate that hypoxia favors the survival of MSCs over that of immune cells and that the expression of fracture healing-relevant genes, most often enhanced by hypoxia, is widely induced. Compared to human *in vitro* and *ex vivo* data and *in vivo* data based on animal models, we could highlight significant similarities. However, further investigations of *ex vivo* equine fracture hematoma are needed to validate our approach and to clarify the cellular and molecular process of the initial phase of fracture healing in the “patient” horse more in detail.

## Supporting information

S1 Appendix File name: Supporting information. (PDF)

## References

[1] Auer JA, Grainger DW. Fracture management in horses: Where have we been and where are we going? Veterinary journal (London, England: 1997). 2015;206(1):5–14. Epub 2015/06/23. 10.1016/j.tvjl.2015.06.002 . 26095036

[2] Rosanowski SM, Chang Y-M, Stirk AJ, Verheyen KLP. Risk factors for race-day fatality in flat racing Thoroughbreds in Great Britain (2000 to 2013). PLOS ONE. 2018;13(3):e0194299 10.1371/journal.pone.0194299 29561898 PMC5862470

[3] Peloso JG, Mundy GD, Cohen ND. Prevalence of, and factors associated with, musculoskeletal racing injuries of thoroughbreds. Journal of the American Veterinary Medical Association. 1994;204(4):620–6. Epub 1994/02/15. . 8163419

[4] Maeda Y, Hanada M, Oikawa M-a. Epidemiology of racing injuries in Thoroughbred racehorses with special reference to bone fractures: Japanese experience from the 1980s to 2000s. Journal of Equine Science. 2016;27(3):81–97. 10.1294/jes.27.81 PMC5048355. 27703403 PMC5048355

[5] Verheyen KL, Wood JL. Descriptive epidemiology of fractures occurring in British Thoroughbred racehorses in training. Equine veterinary journal. 2004;36(2):167–73. Epub 2004/03/25. . 15038441 10.2746/0425164044868684

[6] Janczarek I, Wilk I. Leisure riding horses: research topics versus the needs of stakeholders. Animal science journal = Nihon chikusan Gakkaiho. 2017;88(7):953–8. Epub 2017/04/20. 10.1111/asj.12800 . 28422370

[7] Mejdell CM, Jørgensen GHM, Rehn T, Fremstad K, Keeling L, Bøe KE. Reliability of an injury scoring system for horses. Acta Veterinaria Scandinavica. 2010;52(1):68–. 10.1186/1751-0147-52-68 PMC3023730. 21194451 PMC3023730

[8] Auer JA. Chapter 81—Principles of Fracture Treatment Equine Surgery (Third Edition). Saint Louis: W.B. Saunders; 2006 p. 1000–29.

[9] Auer JA, Watkins JP. Treatment of radial fractures in adult horses: an analysis of 15 clinical cases. Equine veterinary journal. 1987;19(2):103–10. Epub 1987/03/01. . 3569192 10.1111/j.2042-3306.1987.tb02601.x

[10] Jacobs Carrie C, Levine David G, Richardson Dean W. Use of locking compression plates in ulnar fractures of 18 horses*. Veterinary Surgery. 2017;46(2):242–8. 10.1111/vsu.12607 28146292

[11] Turek B, Potynski A, Drewnowska O. Own-design external fixator for the treatment of diaphyseal fractures of the third metacarpal bone in horses. Med Weter. 2016;72(3):197–202. WOS:000371281800010.

[12] Cohen JM, Southwood LL, Engiles J, Leitch M, Nunamaker DM. Effects of a novel hydrogel on equine bone healing: a pilot study. Veterinary and comparative orthopaedics and traumatology: VCOT. 2012;25(3):184–91. Epub 2012/03/01. 10.3415/VCOT-11-01-0006 . 22366873

[13] Govoni KE. HORSE SPECIES SYMPOSIUM: Use of mesenchymal stem cells in fracture repair in horses. Journal of animal science. 2015;93(3):871–8. Epub 2015/05/29. 10.2527/jas.2014-8516 . 26020865

[14] Rosset P, Deschaseaux F, Layrolle P. Cell therapy for bone repair. Orthopaedics & traumatology, surgery & research: OTSR. 2014;100(1 Suppl):S107–12. Epub 2014/01/15. 10.1016/j.otsr.2013.11.010 . 24411717

[15] McDuffee LA, Pack L, Lores M, Wright GM, Esparza-Gonzalez B, Masaoud E. Osteoprogenitor cell therapy in an equine fracture model. Veterinary surgery: VS. 2012;41(7):773–83. Epub 2012/07/19. 10.1111/j.1532-950X.2012.01024.x . 22804243

[16] Murphey ED, Schneider RK, Adams SB, Santschi EM, Stick JA, Ruggles AJ. Long-term outcome of horses with a slab fracture of the central or third tarsal bone treated conservatively: 25 cases (1976–1993). Journal of the American Veterinary Medical Association. 2000;216(12):1949–54. Epub 2000/06/23. . 10863595 10.2460/javma.2000.216.1949

[17] Enneking WF, Burchardt H, Puhl JJ, Piotrowski G. Physical and biological aspects of repair in dog cortical-bone transplants. The Journal of bone and joint surgery American volume. 1975;57(2):237–52. Epub 1975/03/01. . 1089671

[18] Kolar P, Gaber T, Perka C, Duda GN, Buttgereit F. Human early fracture hematoma is characterized by inflammation and hypoxia. Clin Orthop Relat Res. 2011;469(11):3118–26. Epub 2011/03/17. 10.1007/s11999-011-1865-3 21409457 PMC3183184

[19] Schmidt-Bleek K, Schell H, Kolar P, Pfaff M, Perka C, Buttgereit F, et al Cellular composition of the initial fracture hematoma compared to a muscle hematoma: a study in sheep. Journal of orthopaedic research: official publication of the Orthopaedic Research Society. 2009;27(9):1147–51. Epub 2009/04/22. 10.1002/jor.20901 . 19382195

[20] Martini L, Fini M, Giavaresi G, Giardino R. Sheep model in orthopedic research: a literature review. Comparative medicine. 2001;51(4):292–9. Epub 2002/04/02. . 11924786

[21] Holstein JH, Garcia P, Histing T, Kristen A, Scheuer C, Menger MD, et al Advances in the establishment of defined mouse models for the study of fracture healing and bone regeneration. Journal of orthopaedic trauma. 2009;23(5 Suppl):S31–8. Epub 2009/04/29. 10.1097/BOT.0b013e31819f27e5 . 19390374

[22] Seok J, Warren HS, Cuenca AG, Mindrinos MN, Baker HV, Xu W, et al Genomic responses in mouse models poorly mimic human inflammatory diseases. Proceedings of the National Academy of Sciences of the United States of America. 2013;110(9):3507–12. Epub 2013/02/13. 10.1073/pnas.1222878110 23401516 PMC3587220

[23] Pearce AI, Richards RG, Milz S, Schneider E, Pearce SG. Animal models for implant biomaterial research in bone: a review. European cells & materials. 2007;13:1–10. Epub 2007/03/06. . 17334975 10.22203/ecm.v013a01

[24] Marsell R, Einhorn TA. The biology of fracture healing. Injury. 2011;42(6):551–5. Epub 04/13. 10.1016/j.injury.2011.03.031 . 21489527 PMC3105171

[25] Hoff P, Gaber T, Strehl C, Jakstadt M, Hoff H, Schmidt-Bleek K, et al A Pronounced Inflammatory Activity Characterizes the Early Fracture Healing Phase in Immunologically Restricted Patients. International journal of molecular sciences. 2017;18(3). Epub 2017/03/12. 10.3390/ijms18030583 28282868 PMC5372599

[26] Klenke S, Renckhoff K, Engler A, Peters J, Frey UH. Easy-to-use strategy for reference gene selection in quantitative real-time PCR experiments. Naunyn-Schmiedeberg's archives of pharmacology. 2016;389(12):1353–66. Epub 2016/09/22. 10.1007/s00210-016-1305-8 . 27650728

[27] Baddela VS, Baufeld A, Yenuganti VR, Vanselow J, Singh D. Suitable housekeeping genes for normalization of transcript abundance analysis by real-time RT-PCR in cultured bovine granulosa cells during hypoxia and differential cell plating density. Reproductive biology and endocrinology: RB&E. 2014;12:118 Epub 2014/11/29. 10.1186/1477-7827-12-118 25430436 PMC4280684

[28] Kawamoto T, Kawamoto K. Preparation of thin frozen sections from nonfixed and undecalcified hard tissues using Kawamot's film method (2012). Methods in molecular biology (Clifton, NJ). 2014;1130:149–64. Epub 2014/02/01. 10.1007/978-1-62703-989-5_11 . 24482171

[29] Dominici M, Le Blanc K, Mueller I, Slaper-Cortenbach I, Marini FC, Krause DS, et al Minimal criteria for defining multipotent mesenchymal stromal cells. The International Society for Cellular Therapy position statement. Cytotherapy. 2006;8(4):315–7. 10.1080/14653240600855905 WOS:000239953200002. 16923606

[30] Pittenger MF, Mackay AM, Beck SC, Jaiswal RK, Douglas R, Mosca JD, et al Multilineage potential of adult human mesenchymal stem cells. Science. 1999;284(5411):143–7. 10.1126/science.284.5411.143 WOS:000079509000053. 10102814

[31] De Schauwer C, van de Walle GR, Piepers S, Hoogewijs MK, Govaere JL, Meyer E, et al Successful isolation of equine mesenchymal stromal cells from cryopreserved umbilical cord blood-derived mononuclear cell fractions. Equine veterinary journal. 2013;45(4):518–22. Epub 2012/12/05. 10.1111/evj.12003 . 23206252

[32] Maia L, Landim-Alvarenga FC, Da Mota LS, De Assis Golim M, Laufer-Amorim R, De Vita B, et al Immunophenotypic, immunocytochemistry, ultrastructural, and cytogenetic characterization of mesenchymal stem cells from equine bone marrow. Microscopy research and technique. 2013;76(6):618–24. Epub 2013/03/28. 10.1002/jemt.22208 . 23533133

[33] Radtke CL, Nino-Fong R, Gonzalez BPE, Stryhn H, McDuffee LA. Characterization and osteogenic potential of equine muscle tissue- and periosteal tissue-derived mesenchymal stem cells in comparison with bone marrow- and adipose tissue derived mesenchymal stem cells. American journal of veterinary research. 2013;74(5):790–800. WOS:000318203500017. 10.2460/ajvr.74.5.790 23627394

[34] Ranera B, Remacha AR, Alvarez-Arguedas S, Romero A, Vazquez FJ, Zaragoza P, et al Effect of hypoxia on equine mesenchymal stem cells derived from bone marrow and adipose tissue. BMC veterinary research. 2012;8:142 Epub 2012/08/24. 10.1186/1746-6148-8-142 22913590 PMC3483288

[35] Barberini DJ, Freitas NPP, Magnoni MS, Maia L, Listoni AJ, Heckler MC, et al Equine mesenchymal stem cells from bone marrow, adipose tissue and umbilical cord: immunophenotypic characterization and differentiation potential. Stem Cell Research & Therapy. 2014;5(1):25–. 10.1186/scrt414 PMC4055040. 24559797 PMC4055040

[36] Paebst F, Piehler D, Brehm W, Heller S, Schroeck C, Tarnok A, et al Comparative immunophenotyping of equine multipotent mesenchymal stromal cells: an approach toward a standardized definition. Cytometry Part A: the journal of the International Society for Analytical Cytology. 2014;85(8):678–87. Epub 2014/06/05. 10.1002/cyto.a.22491 . 24894974

[37] Treonze KM, Alves K, Fischer P, Hagmann WK, Hora D, Kulick A, et al Characterization of alpha(4)beta(1) (CD49d/CD29) on equine leukocytes: potential utility of a potent alpha(4)beta(1) (CD49d/CD29) receptor antagonist in the treatment of equine heaves (recurrent airway obstruction). Veterinary immunology and immunopathology. 2009;130(1–2):79–87. Epub 2009/03/03. 10.1016/j.vetimm.2009.01.011 . 19250687

[38] De Schauwer C, Piepers S, Van de Walle GR, Demeyere K, Hoogewijs MK, Govaere JL, et al In search for cross-reactivity to immunophenotype equine mesenchymal stromal cells by multicolor flow cytometry. Cytometry Part A: the journal of the International Society for Analytical Cytology. 2012;81(4):312–23. Epub 2012/03/14. 10.1002/cyto.a.22026 . 22411893

[39] Yeo WM, Osterrieder N, Stokol T. Equine herpesvirus type 1 infection induces procoagulant activity in equine monocytes. Veterinary research. 2013;44:16 Epub 2013/03/19. 10.1186/1297-9716-44-16 23497076 PMC3618259

[40] Keramaris NC, Kaptanis S, Moss HL, Loppini M, Pneumaticos S, Maffulli N. Endothelial progenitor cells (EPCs) and mesenchymal stem cells (MSCs) in bone healing. Current stem cell research & therapy. 2012;7(4):293–301. Epub 2012/05/09. . 22563666 10.2174/157488812800793081

[41] Knight MN, Hankenson KD. Mesenchymal Stem Cells in Bone Regeneration. Adv Wound Care (New Rochelle). 2013;2(6):306–16. Epub 2014/02/15. 10.1089/wound.2012.0420 24527352 PMC3842877

[42] Kovach TK, Dighe AS, Lobo PI, Cui Q. Interactions between MSCs and immune cells: implications for bone healing. Journal of immunology research. 2015;2015:752510 Epub 2015/05/23. 10.1155/2015/752510 26000315 PMC4427002

[43] Wagegg M, Gaber T, Lohanatha FL, Hahne M, Strehl C, Fangradt M, et al Hypoxia promotes osteogenesis but suppresses adipogenesis of human mesenchymal stromal cells in a hypoxia-inducible factor-1 dependent manner. PloS one. 2012;7(9):e46483 Epub 2012/10/03. 10.1371/journal.pone.0046483 23029528 PMC3459928

[44] Kovtun A, Bergdolt S, Wiegner R, Radermacher P, Huber-Lang M, Ignatius A. The crucial role of neutrophil granulocytes in bone fracture healing. European cells & materials. 2016;32:152–62. Epub 2016/07/28. . 27452963 10.22203/ecm.v032a10

[45] Grogaard B, Gerdin B, Reikeras O. THE POLYMORPHONUCLEAR LEUKOCYTE—HAS IT A ROLE IN FRACTURE-HEALING. Arch Orthop Trauma Surg. 1990;109(5):268–71. 10.1007/bf00419942 WOS:A1990DV58200007. 2271360

[46] Chung R, Cool JC, Scherer MA, Foster BK, Xian CJ. Roles of neutrophil-mediated inflammatory response in the bony repair of injured growth plate cartilage in young rats. Journal of leukocyte biology. 2006;80(6):1272–80. Epub 2006/09/09. 10.1189/jlb.0606365 . 16959896

[47] Hoff P, Maschmeyer P, Gaber T, Schutze T, Raue T, Schmidt-Bleek K, et al Human immune cells' behavior and survival under bioenergetically restricted conditions in an in vitro fracture hematoma model. Cellular & molecular immunology. 2013;10(2):151–8. Epub 2013/02/12. 10.1038/cmi.2012.56 23396474 PMC4003042

[48] Soehnlein O, Lindbom L, Weber C. Mechanisms underlying neutrophil-mediated monocyte recruitment. Blood. 2009;114(21):4613–23. Epub 2009/08/22. 10.1182/blood-2009-06-221630 . 19696199

[49] Toben D, Schroeder I, El Khassawna T, Mehta M, Hoffmann JE, Frisch JT, et al Fracture Healing Is Accelerated in the Absence of the Adaptive Immune System. J Bone Miner Res. 2011;26(1):113–24. 10.1002/jbmr.185 WOS:000286035600014. 20641004

[50] Reinke S, Geissler S, Taylor WR, Schmidt-Bleek K, Juelke K, Schwachmeyer V, et al Terminally differentiated CD8(+) T cells negatively affect bone regeneration in humans. Science translational medicine. 2013;5(177):177ra36 Epub 2013/03/22. 10.1126/scitranslmed.3004754 . 23515078

[51] de Silly RV, Ducimetiere L, Maroun CY, Dietrich PY, Derouazi M, Walker PR. Phenotypic switch of CD8(+) T cells reactivated under hypoxia toward IL-10 secreting, poorly proliferative effector cells. Eur J Immunol. 2015;45(8):2263–75. 10.1002/eji.201445284 WOS:000359674200012. 25929785 PMC7163737

[52] De Miguel MP, Fuentes-Julian S, Blazquez-Martinez A, Pascual CY, Aller MA, Arias J, et al Immunosuppressive properties of mesenchymal stem cells: advances and applications. Current molecular medicine. 2012;12(5):574–91. Epub 2012/04/21. . 22515979 10.2174/156652412800619950

[53] Faiella W, Atoui R. Immunotolerant Properties of Mesenchymal Stem Cells: Updated Review. Stem cells international. 2016;2016:1859567 Epub 2016/02/04. 10.1155/2016/1859567 26839557 PMC4709780

[54] Duffy MM, Ritter T, Ceredig R, Griffin MD. Mesenchymal stem cell effects on T-cell effector pathways. Stem Cell Res Ther. 2011;2(4):34 Epub 2011/08/25. 10.1186/scrt75 21861858 PMC3219065

[55] Nauta AJ, Fibbe WE. Immunomodulatory properties of mesenchymal stromal cells. Blood. 2007;110(10):3499–506. Epub 2007/08/01. 10.1182/blood-2007-02-069716 . 17664353

[56] Potian JA, Aviv H, Ponzio NM, Harrison JS, Rameshwar P. Veto-like activity of mesenchymal stem cells: functional discrimination between cellular responses to alloantigens and recall antigens. Journal of immunology (Baltimore, Md: 1950). 2003;171(7):3426–34. Epub 2003/09/23. . 14500637 10.4049/jimmunol.171.7.3426

[57] Robey IF, Lien AD, Welsh SJ, Baggett BK, Gillies RJ. Hypoxia-Inducible Factor-1α and the Glycolytic Phenotype in Tumors. Neoplasia (New York, NY). 2005;7(4):324–30. PMC1501147. 10.1593/neo.04430 PMC1501147 15967109

[58] Ito K, Suda T. Metabolic requirements for the maintenance of self-renewing stem cells. Nature reviews Molecular cell biology. 2014;15(4):243–56. Epub 2014/03/22. 10.1038/nrm3772 24651542 PMC4095859

[59] Gaber T, Dziurla R, Tripmacher R, Burmester GR, Buttgereit F. Hypoxia inducible factor (HIF) in rheumatology: low O2! See what HIF can do! Annals of the rheumatic diseases. 2005;64(7):971–80. Epub 2005/04/01. 10.1136/ard.2004.031641 15800008 PMC1755583

[60] Semenza GL. Regulation of mammalian O2 homeostasis by hypoxia-inducible factor 1. Annual review of cell and developmental biology. 1999;15:551–78. Epub 1999/12/28. 10.1146/annurev.cellbio.15.1.551 . 10611972

[61] Liu Y, Berendsen AD, Jia S, Lotinun S, Baron R, Ferrara N, et al Intracellular VEGF regulates the balance between osteoblast and adipocyte differentiation. The Journal of clinical investigation. 2012;122(9):3101–13. Epub 2012/08/14. 10.1172/JCI61209 22886301 PMC3428080

[62] Berendsen AD, Olsen BR. How vascular endothelial growth factor-A (VEGF) regulates differentiation of mesenchymal stem cells. The journal of histochemistry and cytochemistry: official journal of the Histochemistry Society. 2014;62(2):103–8. Epub 2013/12/07. 10.1369/0022155413516347 24309509 PMC3902099

[63] Kottstorfer J, Kaiser G, Thomas A, Gregori M, Kecht M, Domaszewski F, et al The influence of non-osteogenic factors on the expression of M-CSF and VEGF during fracture healing. Injury. 2013;44(7):930–4. Epub 2013/04/11. 10.1016/j.injury.2013.02.028 . 23570706

[64] Ochman S, Frey S, Raschke MJ, Deventer JN, Meffert RH. Local application of VEGF compensates callus deficiency after acute soft tissue trauma—results using a limb-shortening distraction procedure in rabbit tibia. Journal of orthopaedic research: official publication of the Orthopaedic Research Society. 2011;29(7):1093–8. Epub 2011/02/02. 10.1002/jor.21340 . 21284032

[65] Wang CJ, Huang KE, Sun YC, Yang YJ, Ko JY, Weng LH, et al VEGF modulates angiogenesis and osteogenesis in shockwave-promoted fracture healing in rabbits. The Journal of surgical research. 2011;171(1):114–9. Epub 2010/05/11. 10.1016/j.jss.2010.01.045 . 20452608

[66] Schmidt-Bleek K, Schell H, Lienau J, Schulz N, Hoff P, Pfaff M, et al Initial immune reaction and angiogenesis in bone healing. Journal of tissue engineering and regenerative medicine. 2014;8(2):120–30. Epub 2012/04/13. 10.1002/term.1505 . 22495762

[67] Kobayashi T, Onodera S, Kondo E, Tohyama H, Fujiki H, Yokoyama A, et al Impaired fracture healing in macrophage migration inhibitory factor-deficient mice. Osteoporosis international: a journal established as result of cooperation between the European Foundation for Osteoporosis and the National Osteoporosis Foundation of the USA. 2011;22(6):1955–65. Epub 2010/09/15. 10.1007/s00198-010-1385-0 . 20838768

[68] Onodera S, Nishihira J, Yamazaki M, Ishibashi T, Minami A. Increased expression of macrophage migration inhibitory factor during fracture healing in rats. Histochemistry and cell biology. 2004;121(3):209–17. Epub 2004/02/10. 10.1007/s00418-004-0624-x . 14767776

[69] Liu CH, Hwang SM. Cytokine interactions in mesenchymal stem cells from cord blood. Cytokine. 2005;32(6):270–9. Epub 2005/12/27. 10.1016/j.cyto.2005.11.003 . 16377203

[70] Xia W, Xie C, Jiang M, Hou M. Improved survival of mesenchymal stem cells by macrophage migration inhibitory factor. Molecular and cellular biochemistry. 2015;404(1–2):11–24. Epub 2015/02/24. 10.1007/s11010-015-2361-y 25701358 PMC4544672

