# Supplementary material for: Hypoxia and mesenchymal stromal cells as key drivers of initial fracture healing in an equine in vitro fracture hematoma model
Source: PLoS One. 2019 Apr 4;14(4):e0214276. doi: 10.1371/journal.pone.0214276 (PMC6449067; doi:10.1371/journal.pone.0214276)
Supplement: S1 Appendix — (PDF) [file pone.0214276.s001.pdf]

Appendix A

Table A. List of antibodies with their specific reactivity and the dilution used to characterize the cell composition of equine blood and to further characterize equine mesenchymal stromal cells. Cells declared as non-specific were excluded from our study.

Antibody	Reactivity	Dilution	Comments	Refs.
**Rat anti horse CD3:FITC, clone CD3-12, 1 mg/ml, Abcam plc.**	horse-specific	1:20	specific binding, intracellular binding	[1]
**Mouse anti horse CD8:PE, clone CVS21, 1 mg/ml, Bio-Rad AbD Serotec GmbH**	horse-specific	1:20	specific binding	[2]
**Mouse anti horse CD4:FITC, clone CVS4, 1 mg/ml, Bio-Rad AbD Serotec GmbH**	horse-specific	1:500	specific binding	[2]
**Mouse anti horse CD14:APC, clone 105, unknown conc., Cornell University**	horse-specific	1:2.000	specific binding	[3]
**Mouse anti equine Granulocytes:VioBlue, clone HACT39A, 1mg/ml, Kingfisher Biotech Inc.**	horse-specific	1:1.200	specific binding, conjugation in house	-
**Mouse anti horse CD16:PerCP, clone KD1, 1 mg/ml, Abcam plc.**	horse-specific	-	non-specific binding, conjugation in house	[4]
**Mouse anti human CD21, clone Bu33, 1 mg/ml Bio-Rad AbD Serotec GmbH**	cross-reactive	-	non-specific binding	[5]
**Mouse anti horse pan B-Cells:RPE, clone CVS36, 1 mg/ml Bio-Rad AbD Serotec GmbH**	horse-specific	-	non-specific binding	-
**Mouse anti human CD105:Alexa Fluor® 488, clone SN6, 1 mg/ml Bio-Rad AbD Serotec GmbH**	cross-reactive	1:40	specific binding	[6]
**Mouse anti human Integrin beta1/CD29:PE, clone 419127, 25 µg/ml, Bio-Techne GmbH**	cross-reactive	1:20	specific binding	[7]
**Mouse anti horse CD44:RPE, clone CVS18, 1 mg/ml, Bio-Rad AbD Serotec GmbH**	horse-specific	-	non-specific binding	[8]
**Mouse anti horse CD90:DyLight 405®, clone NS1, 1 mg/ml, Abcam plc.**	horse-specific	(-)	specific binding, conjugation in house	[9]

Table B. Depicted is the data of the equine donors both for the blood clots and the *in vitro* fracture hematoma models. For the blood clots the blood of all 4 donors was coagulated (n=4). For the creation of the *in vitro* FH models either blood 1, 2 or 3 was mixed according to the protocol as described above with the MSC of the donor MSC1 (n=3).

Sample Name/Number	Age (years)	Sex	Race
**Blood 1**	6	mare	Trotter
**Blood 2**	14	gelding	Trotter
**Blood 3**	26	mare	Arabian horse
**Blood 4**	22	gelding	Icelander
**MSC 1**	13	gelding	Riding horse

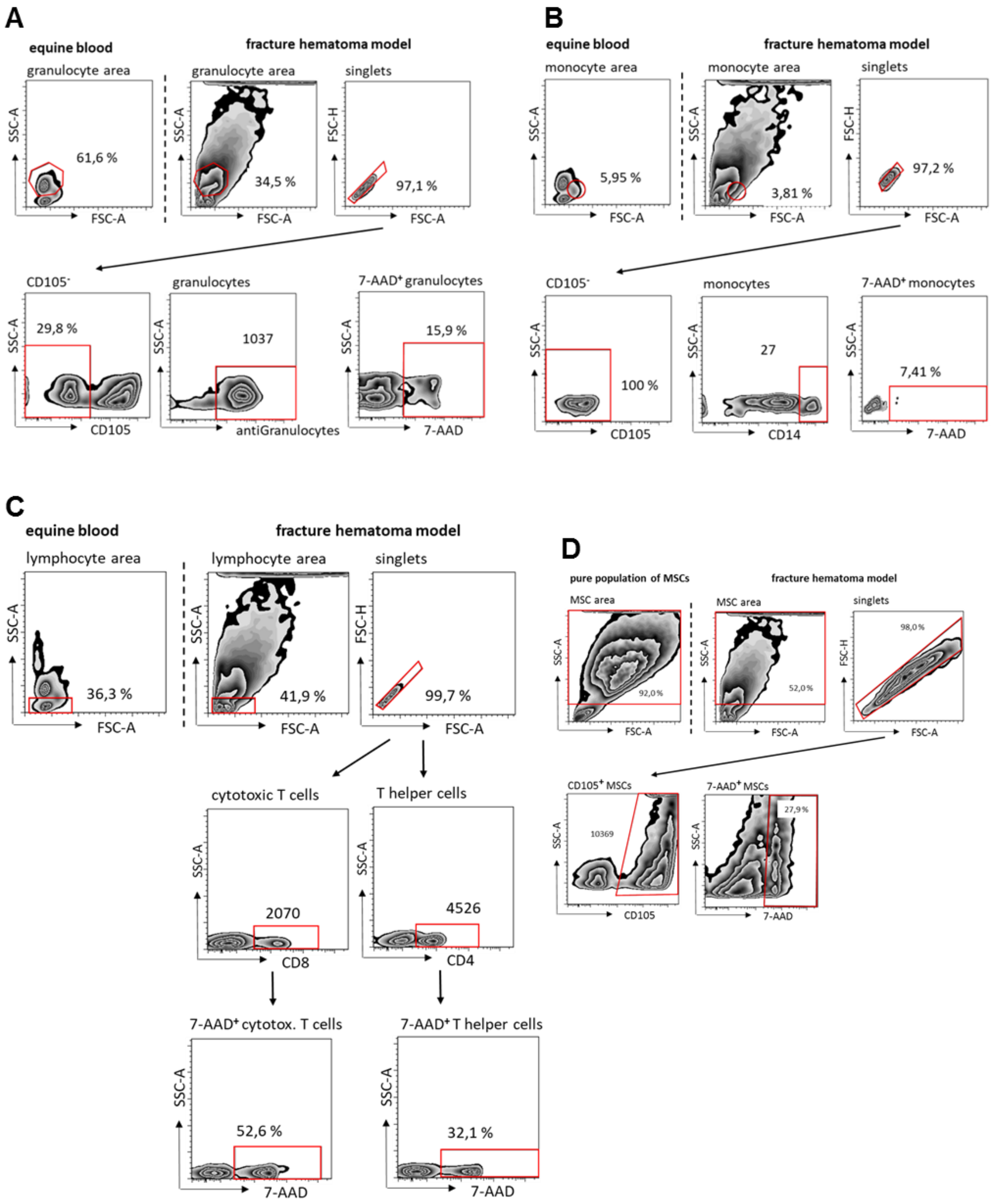

Fig A. Gating strategy for the flow cytometric characterization of cells (MSCs, granulocytes, monocytes, CD4+, and CD8+ cells), within the blood coagulates as well as in the *in vitro* FH models. (A) Gating strategy for the granulocyte population concerning their granularity and size, as well as the anti-granulocyte marker and the absence of the surface marker CD105 (to exclude MSCs). (B) Gating strategy of the monocyte population regarding the expression of CD14 and the lack of expression of CD105. (C) Gating of the lymphocyte fraction and concerning their typical surface markers T-helper-cells (CD4+) and cytotoxic T-cells (CD8+). (D) Gating of the MSC population regarding the typical surface marker CD105.

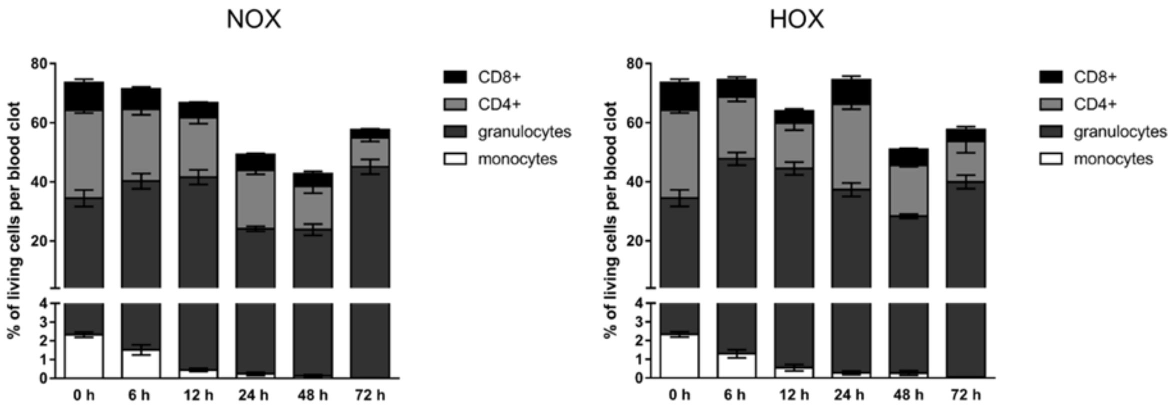

Fig B. Frequency of immune cell populations in blood clots without MSC. Frequency of granulocytes, CD14+ monocytes, CD4+ T cells, CD8+ T cells negative for 7-AAD present in the blood clots as incubated in osteogenic differentiation medium under either hypoxic or normoxic conditions (37 °C, 5% CO2, 18%/ 1% O_2) for 6, 12, 24, 48, and 72 h (mean ± SEM, n = 4).

References

1. Jones M, Cordell JL, Beyers AD, Tse AG, Mason DY. Detection of T and B cells in many animal species using cross-reactive anti-peptide antibodies. Journal of immunology (Baltimore, Md : 1950). 1993;150(12):5429-35. Epub 1993/06/15. PubMed PMID: 8515069.
2. Lunn DP, Holmes MA, Duffus WP. Three monoclonal antibodies identifying antigens on all equine T lymphocytes, and two mutually exclusive T-lymphocyte subsets. Immunology. 1991;74(2):251-7. Epub 1991/10/01. PubMed PMID: 1748472; PubMed Central PMCID: PMCPMC1384601.
3. Yeo WM, Osterrieder N, Stokol T. Equine herpesvirus type 1 infection induces procoagulant activity in equine monocytes. Veterinary research. 2013;44:16. Epub 2013/03/19. doi: 10.1186/1297-9716-44-16. PubMed PMID: 23497076; PubMed Central PMCID: PMCPMC3618259.
4. Elhmouzi-Younes J, Boysen P, Pende D, Storset AK, Le Vern Y, Laurent F, et al. Ovine CD16+/CD14- blood lymphocytes present all the major characteristics of natural killer cells. Veterinary research. 2010;41(1):4. Epub 2009/09/17. doi: 10.1051/vetres/2009052. PubMed PMID: 19755096; PubMed Central PMCID: PMCPMC2762129.
5. Mayall S, Siedek E, Hamblin AS. The anti-human CD21 antibody, BU33, identifies equine B cells. Journal of comparative pathology. 2001;124(1):83-7. Epub 2001/06/29. doi: 10.1053/jcpa.2000.0425. PubMed PMID: 11428193.
6. Braun J, Hack A, Weis-Klemm M, Conrad S, Treml S, Kohler K, et al. Evaluation of the osteogenic and chondrogenic differentiation capacities of equine adipose tissue-derived mesenchymal stem cells. American journal of veterinary research. 2010;71(10):1228-36. Epub 2010/10/06. doi: 10.2460/ajvr.71.10.1228. PubMed PMID: 20919912.
7. Treonze KM, Alves K, Fischer P, Hagmann WK, Hora D, Kulick A, et al. Characterization of alpha(4)beta(1) (CD49d/CD29) on equine leukocytes: potential utility of a potent alpha(4)beta(1) (CD49d/CD29) receptor antagonist in the treatment of equine heaves (recurrent airway obstruction). Veterinary immunology and immunopathology. 2009;130(1-2):79-87. Epub 2009/03/03. doi: 10.1016/j.vetimm.2009.01.011. PubMed PMID: 19250687.
8. De Schauwer C, Piepers S, Van de Walle GR, Demeyere K, Hoogewijs MK, Govaere JL, et al. In search for cross-reactivity to immunophenotype equine mesenchymal stromal cells by multicolor flow cytometry. Cytometry Part A : the journal of the International Society for Analytical Cytology. 2012;81(4):312-23. Epub 2012/03/14. doi: 10.1002/cyto.a.22026. PubMed PMID: 22411893.
9. Marycz K, Kornicka K, Maredziak M, Golonka P, Nicpon J. Equine metabolic syndrome impairs adipose stem cells osteogenic differentiation by predominance of autophagy over selective mitophagy. Journal of cellular and molecular medicine. 2016;20(12):2384-404. Epub 2016/09/16. doi: 10.1111/jcmm.12932. PubMed PMID: 27629697; PubMed Central PMCID: PMCPMC5134411.
